# Activation of Immune Genes in Leafhoppers by Phytoplasmas and Symbiotic Bacteria

**DOI:** 10.3389/fphys.2019.00795

**Published:** 2019-06-21

**Authors:** Elena Gonella, Mauro Mandrioli, Rosemarie Tedeschi, Elena Crotti, Marianna Pontini, Alberto Alma

**Affiliations:** ^1^Dipartimento di Scienze Agrarie, Forestali e Alimentari (DISAFA), Università degli Studi di Torino, Grugliasco, Italy; ^2^Dipartimento di Scienze della Vita (DSV), Università degli Studi di Modena e Reggio Emilia, Modena, Italy; ^3^Dipartimento di Scienze per gli Alimenti, la Nutrizione e l’Ambiente (DeFENS), Università degli Studi di Milano, Milan, Italy

**Keywords:** insect immunity, plant pathogen, symbiotic bacterium, *Asaia*, Raf

## Abstract

Insect immunity is a crucial process in interactions between host and microorganisms and the presence of pathogenic, commensal, or beneficial bacteria may result in different immune responses. In Hemiptera vectors of phytoplasmas, infected insects are amenable to carrying high loads of phytopathogens, besides hosting other bacterial affiliates, which have evolved different strategies to be retained; adaptation to host response and immunomodulation are key aspects of insect-symbiont interactions. Most of the analyses published to date has investigated insect immune response to pathogens, whereas few studies have focused on the role of host immunity in microbiota homeostasis and vectorial capacity. Here the expression of immune genes in the leafhopper vector of phytoplasmas *Euscelidius variegatus* was investigated following exposure to *Asaia* symbiotic bacteria, previously demonstrated to affect phytoplasma acquisition by leafhoppers. The expression of four genes related to major components of immunity was measured, i.e., defensin, phenoloxidase, kazal type 1 serine protease inhibitor and Raf, a component of the Ras/Raf pathway. The response was separately tested in whole insects, midguts and cultured hemocytes. Healthy individuals were assessed along with specimens undergoing early- and late-stage phytoplasma infection. In addition, the adhesion grade of *Asaia* strains was examined to assess whether symbionts could establish a physical barrier against phytoplasma colonization. Our results revealed a specific activation of Raf in midguts after double infection by *Asaia* and flavescence dorée phytoplasma. Increased expression was observed already in early stages of phytoplasma colonization. Gut-specific localization and timing of Raf activation are consistent with the role played by *Asaia* in limiting phytoplasma acquisition by *E. variegatus*, supporting the involvement of this gene in the anti-pathogen activity. However, limited attachment capability was found for *Asaia* under *in vitro* experimental conditions, suggesting a minor contribution of physical phytoplasma exclusion from the vector gut wall. By providing evidence of immune modulation played by *Asaia*, these results contribute to elucidating the molecular mechanisms regulating interference with phytoplasma infection in *E. variegatus*. The involvement of Raf suggests that in the presence of reduced immunity (reported in Hemipterans), immune genes can be differently regulated and recruited to play additional functions, generally played by genes lost by hemipterans.

## Introduction

The interactions of organisms with the surrounding environment are related to their ability in responding to damage and non-self particles. In insects, responses are carried out via innate immunity exclusively, by means of both humoral and cellular defense. A major component of humoral and cellular immune response is the production of antimicrobial peptides (AMPs), which takes place in different organs and tissues, including the digestive tract, salivary glands, and fat body (Tsakas and Marmaras, 2010; Li et al., 2016; Shao et al., 2017). Many AMPs such as defensins are highly conserved in different orders of insects, while others are not evenly distributed (Tedeschi et al., 2017). Moreover, other important components of insect immunity are commonly found, such as phenoloxidase-related machinery and other genes. Insect immune responses are differentially stimulated by either pathogenic or beneficial microorganisms (Tedeschi et al., 2017). Indeed, besides activating responses to neutralize infections by entomopathogens, insects may modulate their immunity in the presence of bacterial symbionts to regulate their density and to maintain the microbiota balance (Login et al., 2011; Eleftherianos et al., 2013; Skidmore and Hansen, 2017). Differently from what is reported for entomopathogens that induce very similar responses, the interaction with non-pathogenic microbes depends on specific traits of insect–microorganism interactions: divergent reactions have indeed been recorded even for closely related microorganisms in the same host. For example, some pathogens of animals or plants, vectored by insects, may establish different interplay with their vectors, which in turn react with different responses. Although pathogenic agents for humans are often perceived as harmful agents by infected insects (Weiss and Aksoy, 2011), in the case of plant pathogens, exclusively transmitted by Hemiptera, divergent kinds of interactions have been observed. Infection by the α-proteobacterial ‘*Candidatus* Liberibacter asiaticus’ resulted in downregulation of immune genes in nymphs of the psyllid *Diaphorina citri* Kuwayama, suggesting that the pathogen is able to modulate the vector immune response to promote its colonization of the hosts (Vyas et al., 2015). Considering plant pathogenic Mollicutes, *Spiroplasma citri* was shown to induce a specific response in the vector *Circulifer haematoceps* (Mulsant and Rey), consisting of increased phagocytosis and upregulation of a gene related to hexamerin, a protein playing a crucial role in phenoloxidase activation (Eliautout et al., 2016). However, the response is balanced by the capability of *S. citri* to inhibit phenoloxidase activity and escape phagocytosis (Eliautout et al., 2016). In phytoplasmas, diverse insect responses have been reported following infection by different strains in the same host species, i.e., immune response or immune priming from infections (Galetto et al., 2018). Moreover, some phytoplasmas counteract the insect response by expressing genes involved in limiting the products of immunity (Makarova et al., 2015).

The leafhopper *Euscelidius variegatus* Kirschbaum (Hemiptera: Cicadellidae) is a polyphagous polyvoltine species capable of transmitting phytoplasmas belonging to different taxonomic groups, including chrysanthemum yellows phytoplasma (CYp, 16SrI group) and flavescence dorée phytoplasma (FDp, 16SrV group), under laboratory conditions. These pathogens have been shown to have opposite effects on *E. variegatus*, with CYp slightly enhancing the fitness of the leafhopper (Bosco and Marzachì, 2016) and FDp reducing its survival and fecundity (Bressan et al., 2005). Transcriptomic analysis of leafhoppers infected with either CYp or FDp has demonstrated that only infection by FDp resulted in activation of insect immune response (Galetto et al., 2018). Besides carrying phytoplasmas, *E. variegatus* harbors bacterial symbionts, like many other Auchenorrhyncha (Baumann, 2005); among these, the acetic acid bacterium *Asaia* has been experimentally documented to limit the acquisition of FDp, after oral administration (Gonella et al., 2018). Symbiont-mediated control mechanisms against phytopathogens include competitive nutrient uptake by symbiotic bacteria, erection of a physical barrier preventing gut establishment and crossing by pathogens, symbiont-mediated immune response of the insect, and the release of antagonistic compounds (Gonella et al., 2019). In *E. variegatus*, the involvement of either *Asaia*-mediated mechanisms stimulating the host immune response or physical exclusion were suggested (Gonella et al., 2018); however, little experimental evidence has been provided on the effects of symbiotic bacteria on *E. variegatus* immunity (Tedeschi et al., 2017) and no data are available on the influence of phytoplasma-symbiont multiple infection on the insect response. Additionally, interest in the molecular machinery involved in the immune response of hemipteran species is hampered by the limited immune repertoire possessed by these insects, as reported for many species (Arp et al., 2016; Skidmore and Hansen, 2017). Such a reduced response is thought to result from the need of Hemiptera to maintain stable relationships with bacterial symbionts. On the other hand, symbiotic bacteria may compensate for the reduction of immunity of their hosts by stimulating insects’ responses to protect them from enemies or directly protecting their hosts from pathogens (Eleftherianos et al., 2013). Moreover, insect immune activation is a candidate strategy used by endosymbionts to modulate the density of other bacteria (Skidmore and Hansen, 2017), including vector-transmitted disease agents, actually altering the insect transmission competence (Weiss and Aksoy, 2011; Kliot et al., 2014). The immune response may be especially crucial for those phytopathogens that cause decreased vector fitness (Cassone et al., 2014; Nachappa et al., 2014; Alma et al., 2015; Olson and Blair, 2015), as in the case of FDp and *E. variegatus*.

In this study, we examined the question as to whether reduced phytoplasma acquisition observed after *Asaia* infection is related to stimulation of the insect immune system, and whether this mechanism is tissue-specific and related to phytoplasma infection timing. To this end, we investigated the expression pattern of four immune genes in *E. variegatus* whole adult leafhoppers, dissected midguts and cultured hemocytes, after exposure to *Asaia* strains and/or FDp. We selected genes involved in different immune pathways to explore possible peculiar regulation of immune genes in insects with reduced immunity such as Hemiptera. In particular, we analyzed phenoloxidase and kazal type 1 serine protease inhibitor, since both genes have been reported to respond to FDp infection (Galetto et al., 2018), together with the *defensin* gene that is one of the most commonly activated genes after bacterial challenges. Moreover, special attention was given to the *Raf* gene, a component of the Ras/Raf pathway, which is known in *Drosophila* as involved in the response to septic injury and in hemocyte proliferation and survival (Asha et al., 2003). The latter was selected since during the latent period (before the insect becomes infective) phytoplasmas multiply in different organs/tissues, including hemocytes, affecting their proliferation/survival (Bosco et al., 2007). Finally, an alternative hypothetical mechanism of interference with FDp acquisition was tested, involving the production of air-liquid interface (ALI) biofilm (Supplementary Table S1), i.e., masking of gut epithelial receptors through adhesion to insect gut wall.

## Materials and Methods

### Insect Material and Bacterial Strains

**FIGURE 1:**
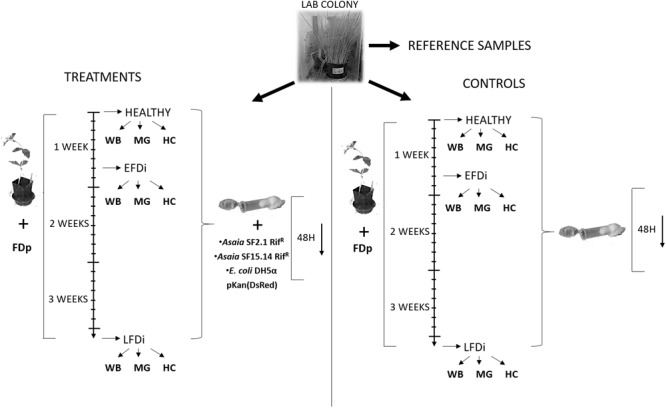
Experimental set up of the trials for expression analysis of immune-related genes. Nymphs of *E. variegatus* were collected from our lab colony as reference samples for gene expression studies, or dedicated to treatments or controls. Insects of the healthy group were directly collected from the lab colony, while those of the EFDi group were exposed to FDp for 5 days and those of the LFDi group were exposed to FDp for 21 days. Afterward, leafhoppers used for treatments were fed either with *Asaia* SF15.14Rif^R^, *Asaia* SF2.1Rif^R^, or *E. coli* DH5α pKan(DsRed) through artificial feeding systems, whereas specimens for the controls were maintained on a sterile sugar diet. Within each group whole insect bodies (WB), midguts (MG), and hemocytes (HC) were taken separately.

A laboratory mass rearing of *E. variegatus* present at the DISAFA laboratories was used as a source of healthy adult leafhoppers for this work. Leafhoppers were kept on oat plants (*Avena sativa* L.) in growth chambers at 25°C with a photoperiod of 16:8 (L:D). Three groups of *E. variegatus* individuals were used for our experiments: healthy adults, specimens at the early stage of phytoplasma colonization, and individuals chronically infected by FDp. The first group was directly collected from the lab colony, while the second and the third groups were obtained by exposing insects to broad beans (*Vicia faba* L.) infected by FDp (strain FD-C). Adults at the early FD phytoplasma infection stage (EFDi) were chosen immediately after being submitted to a 5-day acquisition access period. Late FDp-infected (LFDi) *E. variegatus* were obtained by rearing nymphs on infected broad beans until adult emergence and for at least 21 days (Figure 1).

Bacterial colonization experiments in *E. variegatus* were performed using the spontaneous rifampicin-resistant mutant strains SF2.1Rif^R^ and SF15.14Rif^R^ of *Asaia*, the first being a non-ALI forming strain, and the latter an ALI-producer isolate that was reported to reduce FDp acquisition in *E. variegatus* (Gonella et al., 2018). *Escherichia coli* strain DH5α pKan(DsRed) (Crotti et al., 2009) was also used as a non-symbiotic control. Before use, the strains of *Asaia* were cultivated overnight at 30°C in GLY medium (Favia et al., 2007) under the selection of rifampicin (100 μg/ml), whereas *E. coli* DH5α pKan(DsRed) was cultured overnight at 37°C in Luria-Bertani (LB) medium under the selection of kanamycin (50 μg/ml).

### Bacterial Colonization of *E. variegatus* Individuals

*Asaia* strains SF2.1 Rif^R^ and SF15.14 Rif^R^, as well as *E. coli* DH5α pKan(DsRed), were individually administered to *E. variegatus* adults from the healthy, EFDi, and LFDi groups, following the protocol described by Crotti et al. (2009). Briefly, cultivated bacterial cells were harvested by centrifugation (10 min, 800 *g*), washed three times with 0.9% NaCl, and adjusted to 10^8^ cells/ml in 5% (w/v) sucrose solution in TE (10 mM Tris HCl, 1 mM EDTA, pH 8) (Crotti et al., 2009; Gonella et al., 2012). Insects were allowed to feed for 48 h on artificial feeding systems (Gonella et al., 2015) containing the cell suspensions. Specimens directly collected from the mass rearings without any further treatment were used as reference samples for the gene expression analysis, while adults maintained in the artificial feeding systems with added sterile sugar solution were taken as a control. Five insects for each group and treatment (total number of specimens: 75), corresponding to 5 biological replicates, were directly collected and stored at -80°C in RNA later^®^ (Sigma-Aldrich, St. Louis, MO, United States) until RNA extraction, while further individuals were submitted to midgut dissection.

### Midgut Dissection and Establishment of Hemocyte Cell Cultures

To obtain midgut samples, *E. variegatus* individuals were dissected in Phosphate Buffered Solution (PBS, 137 mM NaCl, 2.7 mM KCl, 10 mM Na_2_HPO_4_, 2 mM KH_2_PO_4_; pH 7.4) made with Diethyl pyrocarbonate-treated water, by using sterile forceps under a stereomicroscope. Five replicates for each insect group and treatment, each one consisting of midguts from three adults, were collected and stored at -80°C in RNA later^®^ (Sigma-Aldrich, St. Louis, MO, United States) until RNA extraction. Dissected midguts from *E. variegatus* adults directly collected from our lab colony without any further treatment were used as reference samples, and the midguts of insects fed with a sterile sugar diet were employed as a control.

Primary hemocyte cell cultures were established following the method described by Tedeschi et al. (2017). Specifically, groups of two adults were washed in 0.115% sodium hypochlorite, 75% ethanol and MilliQ sterile water for 10, 30, and 20 s, respectively, and then dried on a filter paper for a couple of seconds. Washed insects were placed in a single well of a sterile 24-well cell culture plate (Costar^®^, Corning^®^, NY, United States) containing 1 ml of Hert-Hunter 70 medium (Marutani-Hert et al., 2009), supplemented with 10 ml/L L-glutamine (Invitrogen, Carlsbad, CA, United States). Gentamicin (Sigma-Aldrich, St. Louis, MO, United States) at a final concentration of 50 μg/ml, penicillin/streptomycin (Sigma-Aldrich, St. Louis, MO, United States) at a final concentration of 50 U/ml and 50 μg/ml, respectively, and the antimycotic agent nystatin (Sigma-Aldrich, St. Louis, MO, United States) at a final concentration of 100 U/ml, were also added. Plates were incubated at 24 – 26°C for 24 h, to allow cell establishment in the medium, and subsequently incubated for further 24 h with 10^8^ cells/ml bacterial suspension [*Asaia* SF2.1 Rif^R^ and SF15.14 Rif^R^, and *E. coli* DH5α pKan(DsRed)] in the same medium deprived of antibiotics, after removal of the hemocytes culture supernatant. For each of healthy, EFDi and IFDi sample groups, five replicates were treated with bacteria, five replicates were incubated with a sterile antibiotic-free medium in the absence of bacteria as a control, and five replicates were left untreated to be used as the reference samples for gene expression analyses. At the end of treatments, cells were centrifuged at 800 *g* for 5 min at room temperature and immediately subjected to RNA extraction after discarding the supernatant.

### RNA Extraction, cDNA Synthesis, and Quantitative Real Time PCR (qPCR)

RNA extraction was performed with the “SV Total RNA Isolation System” (Promega, Fitchburg, WI, United States), according to the supplier’s suggestions. Briefly, insect tissues were lysed and homogenized with a sterile pestle in 175 μl RNA Lysis Buffer; then samples were heated at 70°C for 3 min after adding 350 μl of RNA Dilution Buffer. Cleared lysate solutions were obtained by centrifugation, and subsequently provided with 200 μl 95% ethanol and transferred in the supplied Spin Column Assembly. Once samples were washed with RNA Wash Solution, they were incubated for 15 min at room temperature with DNase incubation mix, then 200 μl of DNase Stop Solution were added to stop the reaction. Finally, samples were washed twice with RNA Wash Solution and resuspended in 50 μl nuclease-free water. After extraction, RNA quality and concentration were assessed with a ND-1000 spectrophotometer (NanoDrop, Wilmington, DE, United States) and by electrophoresis on a denaturing agarose gel (Supplementary Figure S1). First strand cDNA was synthesized by using the “Reverse Transcription System” (Promega) and Random Primers with 9 μl of RNA, following the manufacturer’s instructions. cDNA was used as a template for qPCR analysis with primer pairs specifically targeting the following genes: *defensin, Raf, phenoloxidase*, and *kazal type 1 serine protease inhibitor*. A list of primers is presented in Table 1. Raf-specific primers were specifically designed on conserved sequences identified by the alignment of Raf sequences of *D. citri* (XM_008488867.2), *Nilaparvata lugens* (Stål) (XM_022347672.1) and *Acyrthosiphon pisum* (Harris) (XM_001952258.4) using the on-line software Primer 3^1^. A preliminary survey was conducted by amplification of cytoplasmic actin with the following parameters: initial denaturation at 95°C for 3 min, then 50 cycles consisting of denaturation at 95°C for 30 s, annealing at 58°C for 40 s and elongation at 72°C for 45 s. Even if a possible influence of immune challenge on actin expression was reported (de-Morais et al., 2005), stable actin expression was observed among sample groups and treatments (Supplementary Table S2), as previously observed by Capone et al. (2013) and Tedeschi et al. (2017). Consequently, *actin* was selected to be amplified as a reference gene. Moreover, samples belonging to the EFDi and LFDi groups were quantitatively checked for FDp infection by 16SrV group phytoplasma-specific PCR reactions, conducted with the fAY/rEY primer pair as described by Galetto et al. (2005); only positive samples were considered in this study. qPCRs were performed on a CFX Connect^TM^ Real-Time PCR Detection System (Bio-Rad, Hercules, CA, United States). Reactions were conducted in clear HardShell^®^ Low-Profile 96-Well PCR Plates (Bio-Rad) with a 25 μl mixture containing 12.5 μl of 2 × SsoFast^TM^ EvaGreen^®^ Supermix (Bio-Rad), 0.1 μl of each primer (100 mM), 100 ng of sample cDNA and 11.3 μl of double distilled H_2_O, sealed with adhesive Microseal^®^ PCR Plate Sealing Film (Bio-Rad); samples were analyzed in triplicate. An initial denaturation at 95°C for 3 min was followed by 50 cycles consisting of denaturation at 95°C for 30 s and annealing at 54°C (for qPCR targeting defensin and Raf) or 58°C (for qPCR targeting phenoloxidase and kazal type 1 serine protease inhibitor) for 20 s. A final step for melting curve analysis from 70 to 95°C, measuring fluorescence every 0.5°C, was added. Results were analyzed using the CFX Manager^TM^ Software (Bio-Rad) for Ct determination. Normalization of primer efficiency was obtained by the one-point calibration (OPC) method, according to Brankatschk et al. (2012); normalized efficiencies of the target genes, with respect to the standard, ranged between 96 and 101%. Relative quantification of target genes was calculated using the 2^-ΔΔCt^ method (Livak and Schmittgen, 2001). Statistical analyses were performed with SPSS Statistics 25 (IBM Corp. Released 2017, Armonk, NY, United States). One-way analysis of variance (ANOVA) was applied and means separated by a Tukey’s test (*P* < 0.05) when variance homogeneity was satisfied (Levene test, *P* < 0.05).

**Table 1:** Primers used in this study.

Primer pair	Target gene	Sequence (5′→3′)	Size (bp)	Source
EvDef F	Defensin	ATGCATTCTTCCATTACTGCTG	200	Tedeschi et al., 2017
EvDef R		CAGCTGCCTCCCTTCTTGC		
Raf F	Raf	CAAGTGGAGAGGATTCAGCAG	200	This study
Raf R		GTGTGTTGGAGCCAGGTCTAT		
PO2_F1020	Phenoloxidase	CAATGTGGTTCCCTCAGGAT	115	Galetto et al., 2018
PO2_R1085		CTGCGAGGTCTCATTTCTGT		
kaz1_F70	Kazal type 1 serine	CTGGTTCGCAGGCAAATACC	103	Galetto et al., 2018
	protease inhibitor
kaz1_R172		GGCATGACACTCGGTACACT		
actF	Actin	AGCAGGAGATGGCCACC	300	Tedeschi et al., 2017
actR		TCCACATCTGCTGGAAGG		
fAY	16S rRNA	GCACGTAATGGTGGGCACTT	300	Marcone et al., 1996
rEY		CGAAGTTAAGCCACTGCTTTC		

### Evaluation of Strain Adhesion Capacity

Adhesion capacity of strains *Asaia* SF2.1Rif^R^, *Asaia* SF15.14Rif^R^, *E. coli* DH5α pKan(DsRed), and the positive control *E. coli* ATCC 25404 was evaluated by crystal violet staining assay (Barbato et al., 2016). *Asaia* strains were grown overnight in GE medium (2% glucose, 0.8% yeast extract, pH 7), while *E. coli* strains were cultured overnight in LB medium [added with 50 μg/ml kanamycin in case of *E. coli* DH5α pKan(DsRed)]. Following overnight growths, 200 μl of the bacterial suspensions containing 10^6^ cell/ml of the different strains were transferred to a flat bottom, polystyrene microtiter plate. Eight replicates were inoculated for each strain and eight uninoculated controls were prepared, as well. Strains were grown at 24 or 30°C for 48 or 72 h. Following the incubation time, the optical density at 610 nm (OD 610 nm) was measured. Bacterial cultures were then removed and microtiter-adhering cells were gently washed with PBS three times. The microtiter plate was dried for 15 min and then stained with a solution of crystal violet (0.5 g/L crystal violet in 20% ethanol) for 15 min at room temperature. Finally, crystal violet solution was removed, the microtiter plate was washed twice with distilled water and let dry for 15 min. Crystal violet contained in adhering cells was then solubilized in 96% ethanol by pipetting. Absorbance (OD 610 nm) of solubilized crystal violet was measured by using a microtiter reader (Tecan Infinite F200Pro).

## Results

### Expression of Immune Genes

**FIGURE 2:**
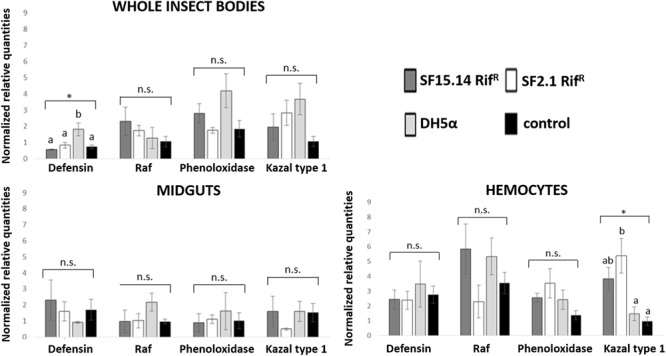
Expression profiles of immune-related genes in *E. variegatus* samples of the healthy group. Gene expression was measured in the whole bodies, midguts, and cultured hemocytes of adults treated with *Asaia* SF15.14Rif^R^, *Asaia* SF2.1Rif^R^, and *E. coli* DH5α pKan(DsRed), along with the control. Normalized relative quantities, calculated by 2^-ΔΔCt^ method, are indicated for *defensin, Raf, phenoloxidase*, and *kazal type 1 serine protease inhibitor* genes. Bars indicate standard errors. Asterisks show significant differences according to ANOVA (*P* < 0.05); different letters indicate significantly different values according to Tukey’s test (a < b). DH5α: *E. coli* DH5α pKan(DsRed); n.s., not significant.

**FIGURE 3:**
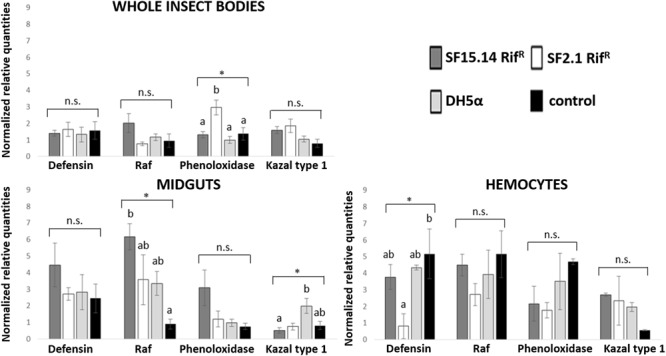
Expression profiles of immune-related genes in *E. variegatus* samples of the EFDi group. Gene expression was measured in the whole bodies, midguts, and cultured hemocytes of adults treated with *Asaia* SF15.14Rif^R^, *Asaia* SF2.1Rif^R^, and *E. coli* DH5α pKan(DsRed), along with the control. Normalized relative quantities, calculated by 2^-ΔΔCt^ method, are indicated for *defensin, Raf, phenoloxidase*, and *kazal type 1 serine protease inhibitor* genes. Bars indicate standard errors. Asterisks show significant differences according to ANOVA (*P* < 0.05); different letters indicate significantly different values according to Tukey’s test (a < b). DH5α: *E. coli* DH5α pKan(DsRed); n.s., not significant.

**FIGURE 4:**
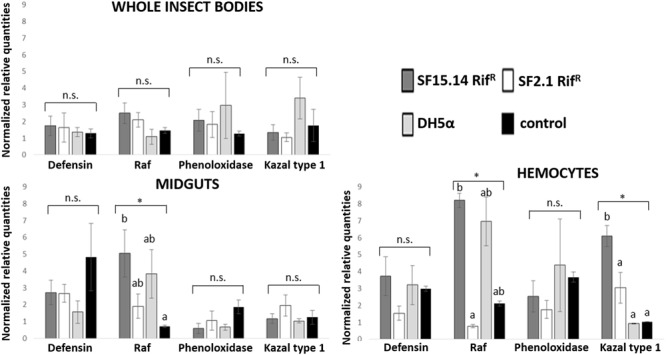
Expression profiles of immune-related genes in *E. variegatus* samples of the LFDi group. Gene expression was measured in the whole bodies, midguts, and cultured hemocytes of adults treated with *Asaia* SF15.14Rif^R^, *Asaia* SF2.1Rif^R^, and *E. coli* DH5α pKan(DsRed), along with the control. Normalized relative quantities, calculated by 2^-ΔΔCt^ method, are indicated for *defensin, Raf, phenoloxidase*, and *kazal type 1 serine protease inhibitor* genes. Bars indicate standard errors. Asterisks show significant differences according to ANOVA (*P* < 0.05); different letters indicate significantly different values according to Tukey’s test (a < b). DH5α: *E. coli* DH5α pKan(DsRed); n.s., not significant.

Prior to measuring the expression of our target immune genes, cDNA from samples belonging to the EFDi and LFDi groups was subjected to phytoplasma quantification to confirm their infection status. We measured the average number of 16SrV phytoplasma cells per sample, which ranged between 1.04 × 10^2^, detected in hemocytes, and 5.54 × 10^4^ found in midguts. No significant differences were observed between treatments within the same sample origin, according to ANOVA performed on log-transformed values (whole insects: df = 7, 32, *F* = 1.466, *P* = 0.215; midguts: df = 7, 32, *F* = 1.042, *P* = 0.422; hemocytes: df = 7, 32, *F* = 1.220, *P* = 0.321). In the EFDi and LFDi sample groups, only the phytoplasma-positive samples were used for the immune gene expression analysis. Normalized relative quantities of immune genes considered in this study are reported in Supplementary Table S3 and Figure 2–4. In the whole bodies of *E. variegatus* adults, the levels of transcripts showed little variability for all genes after supply of either bacteria, in each of the healthy, EFDi, and LFDi groups. Only in a few cases were significant differences observed in the expression values (Supplementary Table S4): defensin was overexpressed in healthy specimens fed with *E. coli* DH5α pKan(DsRed) (Figure 2), while phenoloxidase was activated after administering *Asaia* SF2.1Rif^R^ to individuals of the EFDi group (Figure 3). No differences were detected in LFDi insects (Figure 4). On the other hand, when considering midgut samples, significantly different transcript levels of *Raf* gene were found in the samples exposed to phytoplasmas (EFDi and LFDi groups) (Figure 3, 4). In both cases, Raf was overexpressed in the midguts belonging to specimens fed with *Asaia* SF15.14Rif^R^ relative to the control, consisting of leafhoppers reared in the presence of phytoplasmas without bacteria. *Kazal type 1* gene was found to be upregulated as well, although only in EFDi midguts. In this case, the transcripts related to insects fed with *E. coli* DH5α pKan(DsRed) were significantly more abundant than those detected in the presence of *Asaia* SF15.14Rif^R^. In the case of hemocyte samples, most significant differences were observed for the expression levels of *kazal type 1* gene. Strikingly, upregulation was observed in healthy (Figure 2) and LFDi samples (Figure 4), whereas kazal type 1 transcripts from the EFDi group were not significantly different, particularly when considering hemocytes treated with either bacteria (Figure 3). Moreover, in samples from the healthy group, kazal type 1 was overexpressed after exposure to *Asaia* SF2.1Rif^R^ relative to *E. coli* DH5α pKan(DsRed) and the control, while in samples from the LFDi group the most abundant transcripts were detected in the presence of *Asaia* SF15.14Rif^R^. Other significant differences were recorded in hemocyte samples: defensin was downregulated in samples treated with *Asaia* SF2.1Rif^R^ relative to the control in EFDi samples, and Raf was upregulated in samples exposed to *Asaia* SF15.14Rif^R^ relative to those provided with *Asaia* SF2.1Rif^R^ in samples from the LFDi group (Figure 2–4).

**FIGURE 5:**
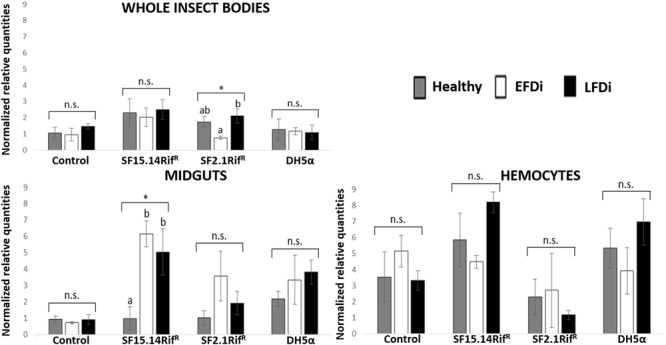
Temporal trend of gene expression for *Raf* gene. Normalized relative quantities are displayed for whole insect bodies, midgut sections, and cultured hemocytes of insects treated with *Asaia* SF15.14Rif^R^, *Asaia* SF2.1Rif^R^, and *E. coli* DH5α pKan(DsRed), along with the control. Healthy: time 0 of FDp infection; EFDi time 0 + 5 days of FDp infection; LFDi: time 0 + 21 days of FDp infection. Asterisks show significant differences according to ANOVA (*P* < 0.05); different letters indicate significantly different values according to Tukey’s test (a < b). DH5α: *E. coli* DH5α pKan(DsRed); n.s., not significant.

**FIGURE 6:**
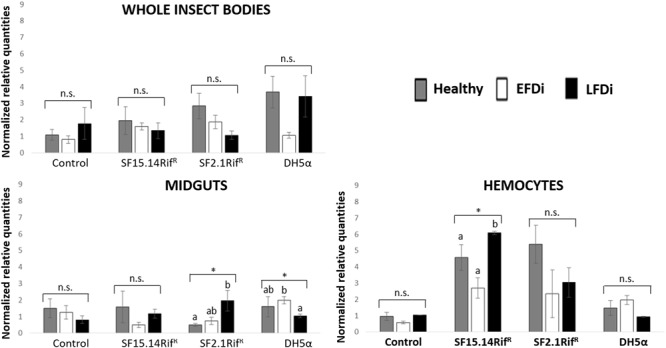
Temporal trend of gene expression for *kazal type 1 serine protease inhibitor* gene. Normalized relative quantities are displayed for whole insect bodies, midgut sections, and cultured hemocytes of insects treated with *Asaia* SF15.14Rif^R^, *Asaia* SF2.1Rif^R^, and *E. coli* DH5α pKan(DsRed), along with the control. Healthy: time 0 of FDp infection; EFDi time 0 + 5 days of FDp infection; LFDi: time 0 + 21 days of FDp infection. Asterisks show significant differences according to ANOVA (*P* < 0.05); different letters indicate significantly different values according to Tukey’s test (a < b). DH5α: *E. coli* DH5α pKan(DsRed); n.s., not significant.

**FIGURE 7:**
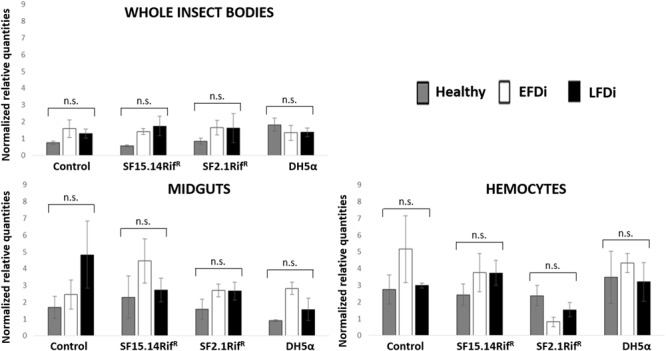
Temporal trend of gene expression for *defensin* gene. Normalized relative quantities are displayed for whole insect bodies, midgut sections, and cultured hemocytes of insects treated with *Asaia* SF15.14Rif^R^, *Asaia* SF2.1Rif^R^, and *E. coli* DH5α pKan(DsRed), along with the control. Healthy: time 0 of FDp infection; EFDi time 0 + 5 days of FDp infection; LFDi: time 0 + 21 days of FDp infection. DH5α: *E. coli* DH5α pKan(DsRed); n.s., not significant.

**FIGURE 8:**
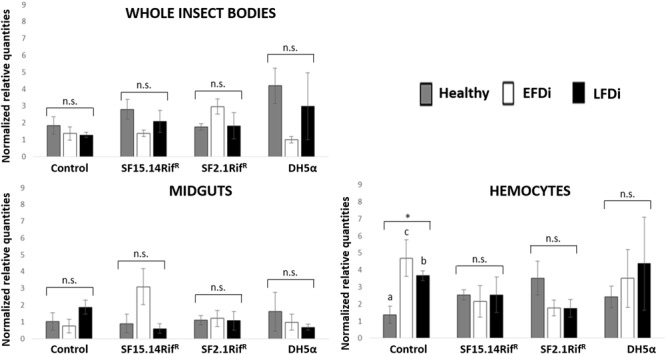
Temporal trend of gene expression for *phenoxidase* gene. Normalized relative quantities are displayed for whole insect bodies, midgut sections, and cultured hemocytes of insects treated with *Asaia* SF15.14Rif^R^, *Asaia* SF2.1Rif^R^, and *E. coli* DH5α pKan(DsRed), along with the control. Healthy: time 0 of FDp infection; EFDi time 0 + 5 days of FDp infection; LFDi: time 0 + 21 days of FDp infection. Asterisk shows significant difference according to ANOVA (*P* < 0.05); different letters indicate significantly different values according to Tukey’s test (a < b). DH5α: *E. coli* DH5α pKan(DsRed); n.s., not significant.

Besides comparing gene expression profiles obtained for different treatments and sample groups, data were further analyzed to evaluate the temporal expression trend of each gene with respect to phytoplasma infection. Hence, normalized values obtained for single genes were examined, considering the healthy group as time 0 with respect to FDp infection, the EFDi group as time 0 + 5 days from the beginning of FDp infection, and finally the LFDi group as time 0 + 21 days from the beginning of phytoplasma infection. Statistical analysis performed to compare data from similarly treated samples from the healthy, EFDi and LFDi groups revealed a growing expression trend only for *Raf* and *kazal type 1* genes (Figure 5, 6). Specifically, Raf was overexpressed in the whole body of *E. variegatus* fed with *Asaia* SF2.1Rif^R^ as a consequence of chronical FDp infection (LFDi vs. healthy + EFDi), and in the midguts of insects provided with *Asaia* SF15.14Rif^R^ subsequent to FDp exposure (EFDi + LFDi vs. healthy) (Figure 5 and Supplementary Table S5). Likewise, *kazal type 1* gene was upregulated in samples from the LFDi group (LFDi vs. healthy + EFDi), and precisely in midguts of insects fed with *Asaia* SF2.1Rif^R^ and *E. coli* DH5α pKan(DsRed), and in hemocytes following treatment with *Asaia* SF15.14Rif^R^ (Figure 6 and Supplementary Table S5). Conversely, we did not find any significant trend in defensin or phenoloxidase in consequence of bacterial challenge (Figure 7, 8 and Supplementary Table S5). Instead, phenoloxidase was significantly overexpressed over time in hemocytes from the control group (Figure 8).

### Adhesion Test

Adhesion capacity of *Asaia* strains was evaluated in comparison to the one shown by a well-known biofilm-producer strain, i.e., *E. coli* ATCC 25404, which was considered as positive control (Wood et al., 2006). *Asaia* SF2.1Rif^R^ and *E. coli* DH5α pKan(DsRed) weakly adhered to the polystyrene microtiter plate (Supplementary Figure S2A and Supplementary Table S6) in comparison with the positive control strain. Moreover, following a longer incubation (72 h) *E. coli* DH5α pKan(DsRed) displayed a decreasing adhesion capacity. *Asaia* SF15.14Rif^R^ was not able to adhere to the microtiter wells in our experimental conditions either considering incubation at 24 and 30°C, or 48 and 72 h. In wells inoculated with *Asaia* SF15.14Rif^R^ bacterial biomasses were observed on the medium surface that resembled the ALI films already described to be produced by this strain (Supplementary Figure S2B): it is likely that the shorter incubation times used in our experiments, in comparison to those described in Gonella et al. (2018) did not allow the complete film formation observed before. Conversely, strain *E. coli* ATCC 25404 showed itself to be a strong adherent strain.

## Discussion

Our investigation focused on the differential regulation of immune genes chosen to represent a range of elements shaping the leafhopper immune response, revealing localized regulation in *E. variegatus* adults, for all of the four genes taken into consideration. Indeed, even if gene expression variability generally appeared mitigated in whole-body samples for most of the genes, in midgut and hemocyte samples, significantly diverging gene expression levels were observed, in particular for the genes *Raf* and *kazal type 1*.

*Raf* is the only gene that exhibited consistent upregulation in response to exposure to a single bacterial strain, being overexpressed in the presence of *Asaia* SF15.14Rif^R^. Since this strain was reported by Gonella et al. (2018) to induce a reduced FDp acquisition in *E. variegatus*, this gene might be involved in enhancing the insect response to phytoplasma infection. Specifically, *Raf* gene was more specifically activated in midgut samples when the presence of *Asaia* SF15.14Rif^R^ was combined with FDp infection (EFDi and LFDi groups), while the same gene was overexpressed in the hemocytes only at the late phase of phytoplasma infection. Our experimental evidence confirms that Raf expression is relevant in the immune response and in persistent infection and/or sepsis since Raf induction causes activation of the hematopoiesis (as reported in *Drosophila*) (Asha et al., 2003; Zettervall et al., 2004). Our observations also suggest that the midgut could be a crucial site for Raf activation, supporting a role for Raf in limiting the capability of FDp to colonize the gut, and crossing this barrier to reach the hemolymph. This indication is in accordance with previous results suggesting that gut homeostasis is maintained through a balance between cell damage, due to the collateral effects of bacteria killing, and epithelial repair by stem cell division (Buchon et al., 2009) and that Raf is involved in the control of *Drosophila* gut stem cell proliferation (Jin et al., 2015). Accordingly, in the midgut of insects fed with *Asaia* SF15.14Rif^R^, a significant rise of Raf expression was recorded corresponding to the early stage of FDp infection, which is a crucial stage for phytoplasma passage from the midgut to the insect hemolymph.

In the case of kazal type 1 serine protease inhibitor, a hemocyte-specific response was observed following challenge with *Asaia*, while no significant activation relative to controls was observed in whole insects or dissected midguts following exposure to these strains. Nonetheless, different strains induced kazal type 1 overexpression in the hemocytes of healthy and LFDi individuals, namely strains SF2.1Rif^R^ and SF15.14Rif^R^ in the first and in the latter groups, respectively, whereas no significant upregulation was found in the EFDi hemocytes. Accordingly, significantly increased expression of kazal type 1 was found in late phytoplasma infection (LFDi), in hemocytes exposed to *Asaia* SF15.14Rif^R^ (while an increasing trend over FDp infection time was observed for strain SF2.1Rif^R^ in midgut samples). Hence, a response in the hemocytes could be speculated to be related to genus-specific traits rather than to the production of ALI biofilm. Previous work experimentally combing *Asaia* strains with FDp infection did not show inhibition of phytoplasma transmission by strain SF2.1Rif^R^ (Gonella et al., 2018), suggesting the absence of genus-specific interference with the pathogen. Moreover, although FDp itself was demonstrated to induce *kazal type 1* gene overexpression (Galetto et al., 2018), this pathogen can bloom to high concentrations in *E. variegatus* (Rashidi et al., 2014), indicating that phytoplasmas may be only partially susceptible to such a response. Furthermore, no clear temporal trend was observed for *kazal type 1* gene over FDp infection stages in the control group, in none of sample types, even though a slight increment was visible for whole-insect samples, in agreement with the upregulation reported by Galetto et al. (2018). This is indicative of limited activation of this gene in our experimental conditions. Taken together, this evidence does not support a role for kazal type 1 activation in limiting FDp acquisition by *E. variegatus*.

The expression of *defensin* and *phenoloxidase* genes was not as affected as that of *Raf* and *kazal type 1* genes in response to bacterial infection. Defensin was overexpressed in the whole body of insects fed with *E. coli* DH5α pKan(DsRed) only considering healthy individuals. Conversely, considering the EFDi and LFDi insect groups and single-body parts, samples exposed to this strain did not exhibit higher defensin expression than those treated with *Asaia* strains or the control, confirming the limited activation of this AMP in *E. variegatus* in response to Gram-negative bacteria (Tedeschi et al., 2017). Relative defensin transcript levels were significantly reduced in the hemocytes of insects from the EFDi group, after challenge with *Asaia* SF2.1Rif^R^. However, no further data sustained the downregulation of this gene operated by the non-ALI producer strain. In previous experiments performed with hemocyte cultures of *Anopheles stephensi* Liston and *Drosophila melanogaster* Meigen, the administration of a DsRed-tagged strain of *Asaia* SF2.1 and *E. coli* DH5α pKan(DsRed) did not induce the expression of *defensin* gene (Capone et al., 2013). However, it is worth considering that a higher load of bacterial cells (10^9^ cell/ml) and shorter incubation times (0, 4, 8, 12 h) than the ones used in our experimental set up were used by Capone et al. (2013). Taking into account the tendency of defensin expression over FDp infection-time, statistical analysis did not demonstrate any significant variation. However, an increase in the expression levels was recorded in the midgut of control insects, never exposed to bacteria other than the phytoplasma. This is in agreement with evidence reported by Yang et al. (2017), showing that defensin is activated by insect infection with *Spiroplasma melliferum*, a close relative of phytoplasmas recognized as a model for phytoplasma infection (Naor et al., 2011). On the other hand, when analyzing the FDp infection temporal trend in the control hemocytes, enhanced expression was detected in correspondence to the early stage of pathogen infection, suggesting hemocyte-specific defensin stimulation, consistently with the key role of the hemolymph for phytoplasma multiplication, resulting in a higher load of phytopathogen cells, which in turn stimulated an insect response. However, our data suggest an overall limited role of defensin in altering the capacity of *E. variegatus* to acquire FDp.

Phenoloxidase exhibited mostly irregular expression profiles, without significant diversity of transcript levels corresponding to distinct bacterial treatments according to ANOVA analysis, with the only exception being EFDi whole insects. Such an erratic response suggests the absence of a specific induction mechanism related to a challenge with any of administered bacterial strains. Limited phenoloxidase activation in response to *Asaia* infection could be expected considering its symbiotic interplay with leafhoppers (Crotti et al., 2009). On the other hand, in *Anopheles* mosquitoes, the adaptation of *Asaia* to host body environment was suggested to be related to resistance to immune response, rather than to a low immunogenicity (Capone et al., 2013); however, these authors did not investigate phenoloxidase activation. Similarly, although being a non-symbiotic strain, *E. coli* DH5α pHM2(GFP) did not induce phenoloxidase-related responses in leafhoppers, as previously reported for *E. coli*-infected hemocytes of *C. haematoceps* (Eliautout et al., 2016). However, phenoloxidase was shown to be significantly activated in the hemocytes during the early stage of phytoplasma infection, as a transcript peak was detected for EFDi samples in the control group. This result supports the hypothesis put forward by Galetto et al. (2018) that an increment in the expression of phenoloxidase might be evident in the early stages of FDp infection, whereas a lack of activation is typical of *E. variegatus* individuals chronically infected by phytoplasma.

Besides demonstrating that *Asaia* SF15.14Rif^R^ elicited in *E. variegatus* a midgut-specific immune response, this work explored an alternative process that may modulate the interference exerted by this strain on FDp, i.e., physical exclusion by means of complete adhesion to the gut wall. However, the adhesion test did not show successful adhesion to microtiter plates for *Asaia* SF15.14Rif^R^. This result might be affected by the experimental conditions that were applied in terms of incubation times or medium used. The artificial substrate may indeed imperfectly mimic the insect gut epithelia. As a matter of fact, in Hemiptera the gut content is in contact with the microvilli of midgut cells, in the absence of a peritrophic membrane (Nation, 2016). Therefore, the increased contact surface provided by microvilli may offer a more suitable substrate for bacterial adhesion. Acetic acid bacteria closely related to *Asaia* were reported to be specifically located near the host gut wall (Kounatidis et al., 2009; Vacchini et al., 2017); however, our results do not support massive attachment of *Asaia* SF15.14Rif^R^ to the gut epithelial layer. A possible explanation of limited attachment could be the production of flocculant flake-like bacterial masses (Supplementary Figure S2B). These masses have previously been proposed to play a role in entrapping phytoplasma cells or erecting a barrier against them, contributing to hampering pathogen establishment in the insect body (Gonella et al., 2018). Moreover, we cannot rule out the involvement of specific host factors under *in vivo* conditions necessary to mediate the bacterial adhesion. Future work is thus required to investigate the role of bacterial entrapment or physical containment in limiting FDp acquisition by *E. variegatus*.

## Conclusion

This work sheds light on the molecular interplay occurring among insect hosts and bacteria, focusing on symbiotic and non-symbiotic strains, as well as on leafhopper-vectored plant pathogens. Among different immune-related genes indicative of distinct response mechanisms, *Raf* gene showed midgut-specific activation in response to *Asaia* strain SF15.14Rif^R^ after insect infection by FDp, while the pathogen alone did not stimulate the same reaction. It can therefore be suggested that *Asaia* strain SF15.14Rif^R^ may elicit a basal host immune activity that appears to act against other microorganisms, including phytoplasma. The elicitation of Raf-mediated response is thus predicted to be a major component of the interfering effect displayed by *Asaia* SF15.14Rif^R^ toward FDp acquisition by *E. variegatus*. Considering the limited number of immune genes reported for *E. variegatus* and other Hemiptera (Arp et al., 2016; Skidmore and Hansen, 2017; Tedeschi et al., 2017), our results suggest a compensation of lost defensive functions by genes that have minor functions in insects exhibiting a more complete immunity. Investigating the role of other immune genes will help to further elucidate this phenomenon, as well as exploring possible differential response profiles in different conditions, such as for example when insects are exposed to different temperatures. On the other hand, a minor contribution to phytoplasma exclusion seems to be provided by *Asaia* attachment to the vector gut wall. Further possible mechanisms of phytoplasma segregation, involving a physical barrier created by *Asaia* SF15.14Rif^R^ though the production of ALI biofilm, need to be investigated.

## Author Contributions

EG, MM, RT, EC, and AA contributed to conception and design of the study. EG, EC, and MP carried out the experiments. EG performed the statistical analysis. AA, RT, and EC provided reagents and analytical tools. EG, MM, EC, and RT wrote sections of the manuscript. All authors contributed to manuscript revision, read and approved the submitted version.

## Conflict of Interest Statement

The authors declare that the research was conducted in the absence of any commercial or financial relationships that could be construed as a potential conflict of interest.

## Footnotes

1 http://bioinfo.ut.ee/primer3/
