# Supplementary material for: Activation of Immune Genes in Leafhoppers by Phytoplasmas and Symbiotic Bacteria
Source: Front Physiol. 2019 Jun 21;10:795. doi: 10.3389/fphys.2019.00795 (PMC6598074; doi:10.3389/fphys.2019.00795)
Supplement: Supplementary file 1 [file Table_1.docx]

SUPPLEMENTARY INFORMATION

Activation of immune related genes in leafhoppers by phytoplasmas and symbiotic bacteria

Elena Gonella^1^, Mauro Mandrioli^2^, Rosemarie Tedeschi^1^, Elena Crotti^3^, Marianna Pontini^1^, Alberto Alma^1*^

^1^Dipartimento di Scienze Agrarie, Forestali e Alimentari (DISAFA), Università degli Studi di Torino, Grugliasco, Italy

^2^Dipartimento di Scienze della Vita (DSV), Università degli Studi di Modena e Reggio Emilia, Modena, Italy

^3^Dipartimento di Scienze per gli Alimenti, la Nutrizione e l’Ambiente (DeFENS), Università degli Studi di Milano, Milano, Italy

*** Correspondence:**Alberto Alma
alberto.alma@unito.it

**SUPPLEMENTARY TABLES**

**TABLE S1** List of Abbreviations used in this paper.

| **Abbreviation** | **Extended term** |
| --- | --- |
| AMP | AntiMicrobial Peptides |
| CYp | Chrysanthemum Yellows Phytoplasma |
| FDp | Flavescence Dorée Phytoplasma |
| ALI | Air-Liquid Interface |
| DISAFA | DIpartimento di Scienze Agrarie, Forestali e Alimentari (University of Torino) |
| EFDi | Early Flavescence Dorée Infection |
| LFDi | Late Flavescence Dorée Infection |
| PBS | Phosphate Buffered Saline |
| LB | Luria Bertani |

**Table S2**. QPCR stability values, calculated using NormFinder, indicated that actin gene was expressed stably with respect to each sample type and treatment.

| **Whole insect bodies** | | **Midguts** | | **Hemocytes** | |
| --- | --- | --- | --- | --- | --- |
| **Treatment** | **Stability value** | **Treatment** | **Stability value** | **Treatment** | **Stability value** |
| Reference samples | 0.019 | Reference samples | 0.031 | Reference samples | 0.036 |
| Healthy Control | 0.056 | Healthy Control | 0.075 | Healthy Control | 0.023 |
| EFDi Control | 0.021 | EFDi Control | 0.042 | EFDi Control | 0.057 |
| LFDi Control | 0.017 | LFDi Control | 0.037 | LFDi Control | 0.040 |
| Healthy SF15.14Rif^R^ | 0.067 | Healthy SF15.14Rif^R^ | 0.043 | Healthy SF15.14Rif^R^ | 0.027 |
| EFDi SF15.14Rif^R^ | 0.041 | EFDi SF15.14Rif^R^ | 0.016 | EFDi SF15.14Rif^R^ | 0.042 |
| LFDi SF15.14Rif^R^ | 0.047 | LFDi SF15.14Rif^R^ | 0.026 | LFDi SF15.14Rif^R^ | 0.023 |
| Healthy SF2.1Rif^R^ | 0.020 | Healthy SF2.1Rif^R^ | 0.030 | Healthy SF2.1Rif^R^ | 0.030 |
| EFDi SF2.1Rif^R^ | 0.026 | EFDi SF2.1Rif^R^ | 0.064 | EFDi SF2.1Rif^R^ | 0.050 |
| LFDi SF2.1Rif^R^ | 0.067 | LFDi SF2.1Rif^R^ | 0.028 | LFDi SF2.1Rif^R^ | 0.036 |
| Healthy DH5α | 0.044 | Healthy DH5α | 0.039 | Healthy DH5α | 0.047 |
| EFDi DH5α | 0.068 | EFDi DH5α | 0.015 | EFDi DH5α | 0.052 |
| LFDi DH5α | 0.077 | LFDi DH5α | 0.022 | LFDi DH5α | 0.040 |

**TABLE S3** Normalized relative quantities of *defensin, Raf, phenoloxidase* and *kazal type 1 serine protease inhibitor* genes obtained in this study. SE: Standard Error; DH5α: *E. coli* DH5α pKan(DsRed).

| **Treatment** | **Mean normalized relative quantity (±SE)** | | | |
| --- | --- | --- | --- | --- |
|  | *defensin* | *Raf* | *phenoloxidase* | *kazal type 1 serine protease inhibitor* |
| **WHOLE INSECT BODIES** |  |  |  |  |
| Healthy Control | 0.736±0.101 | 1.064±0.330 | 1,846±0.518 | 1.073±0.320 |
| EFDi Control | 1.577±0.533 | 0.955±0.404 | 1.370±0.386 | 0.793±0.239 |
| LFDi Control | 1.290±0.276 | 1.464±0.165 | 1.277±0.146 | 1.7690±0.961 |
| Healthy SF15.14Rif^R^ | 0.558±0.048 | 2.308±0.861 | 2.802±0.592 | 1.944±0.840 |
| EFDi SF15.14Rif^R^ | 1.406±0.174 | 2.026±0.572 | 1.308±0.192 | 1.595±0.205 |
| LFDi SF15.14Rif^R^ | 1.738±0.579 | 2.506±0.608 | 2.084±0.656 | 1.337±0.469 |
| Healthy SF2.1Rif^R^ | 0.835±0.177 | 1.745±0.322 | 1.758±0.185 | 2.836±0.782 |
| EFDi SF2.1Rif^R^ | 1.644±0.426 | 0.767±0.100 | 2.960±0.451 | 1.864±0.405 |
| LFDi SF2.1Rif^R^ | 1.628±0.869 | 2.111±0.436 | 1.818±0.771 | 1.054±0.256 |
| Healthy DH5α | 1.812±0.393 | 1.284±0.650 | 4.195±1.054 | 3.678±0.967 |
| EFDi DH5α | 1.338±0.449 | 1.166±0.211 | 0.999±0.198 | 1.056±0.190 |
| LFDi DH5α | 1.365±0.265 | 1.088±0.454 | 2.971±1.984 | 3.414±1.255 |
| **MIDGUTS** |  |  |  |  |
| Healthy Control | 1.704±0.648 | 0.949±0.155 | 1.016±0.510 | 1.516±0.573 |
| EFDi Control | 2.450±0.875 | 0.902±0.299 | 0.756±0.191 | 0.807±0.454 |
| LFDi Control | 4.825±1.999 | 0.712±0.065 | 1.869±0.412 | 1.255±0.400 |
| Healthy SF15.14Rif^R^ | 2.308±1.252 | 0.975±0.708 | 0.892±0.564 | 1.597±0.950 |
| EFDi SF15.14Rif^R^ | 4.461±1.316 | 6.168±0.792 | 3.093±1.074 | 0.509±0.165 |
| LFDi SF15.14Rif^R^ | 2.728±0.715 | 5.049±1.403 | 1.052±0.290 | 1.288±0.275 |
| Healthy SF2.1Rif^R^ | 1.591±0.597 | 1.013±0.445 | 1.100±0.274 | 0.500±0.073 |
| EFDi SF2.1Rif^R^ | 2.710±0.374 | 3.571±1.514 | 1.203±0.475 | 0.750±0.211 |
| LFDi SF2.1Rif^R^ | 2.670±0.529 | 1.914±0.720 | 1.062±0.563 | 1.967±0.625 |
| Healthy DH5α | 0.915±0.053 | 2.179±0.568 | 1.612±1.151 | 1.602±0.618 |
| EFDi DH5α | 2.823±1.048 | 3.348±0.722 | 0.976±0.142 | 1.988±0.483 |
| LFDi DH5α | 1.570±0.669 | 3.825±1.440 | 0.672±0.178 | 1.045±0.112 |
| **HEMOCYTES** |  |  |  |  |
| Healthy Control | 2.757±0.875 | 3.546±1.556 | 1.377±0.510 | 0.965±0.239 |
| EFDi Control | 5.167±1.490 | 5.149±1.420 | 4.694±0.177 | 0.577±0.025 |
| LFDi Control | 2.987±0.147 | 2.118±0.153 | 3.674±0.290 | 1.032±0.003 |
| Healthy SF15.14Rif^R^ | 2.438±0.629 | 5.850±1.678 | 2.546±0.297 | 3.823±0.784 |
| EFDi SF15.14Rif^R^ | 3.761±0.748 | 4.489±0.654 | 2.158±1.045 | 2.705±0.101 |
| LFDi SF15.14Rif^R^ | 3.738±1.142 | 8.207±0.416 | 2.545±0.926 | 6.091±0.625 |
| Healthy SF2.1Rif^R^ | 2.382±0.620 | 2.297±1.109 | 3.528±0.997 | 5.391±1.166 |
| EFDi SF2.1Rif^R^ | 0.814±0.279 | 2.711±2.313 | 1.771±0.464 | 2.352±1.466 |
| LFDi SF2.1Rif^R^ | 1.538±0.420 | 0.781±0.089 | 1.751±0.527 | 3.039±0.919 |
| Healthy DH5α | 3.478±1.556 | 5.340±1.231 | 2.421±0.642 | 1.474±0.452 |
| EFDi DH5α | 4.337±0.569 | 3.929±1.447 | 3.507±1.697 | 1.961±0.278 |
| LFDi DH5α | 3.208±1.161 | 6.9728753±3.592 | 4.383±2.733 | 0.936±0.029 |

**TABLE S4** Results of statistical analysis (ANOVA + Tukey’s test, P < 0.05) performed to compare the expression levels of the immune-related genes after colonization with *Asaia* SF15.14Rif^R^, *Asaia* SF2.1Rif^R^, *E. coli* DH5α pKan(DsRed), and control. *Defensin, Raf, phenoloxidase* and *kazal type 1 serine protease inhibitor* genes were analysed in in *E. variegatus* whole insects, dissected midguts, and hemocytes. Healthy insects were studied along with individuals at the early stage of FDp infection (EFDi), as well and chronically infected leafhoppers (LFDi). Degrees of Freedom: 3 (among-group), 16 (within-group).

| **Sample group** | **Sample type** | **Target gene** | **F-value** | **P-value** |
| --- | --- | --- | --- | --- |
| **Healthy** | Whole insect bodies | defensin | 6.374 | <0.05 |
|  |  | Raf | 0.881 | 0.472 |
|  |  | phenoloxidase | 2.912 | 0.067 |
|  |  | kazal type 1 | 2.146 | 0.135 |
|  | Midguts | defensin | 0.287 | 0.834 |
|  |  | Raf | 1.766 | 0.194 |
|  |  | phenoloxidase | 1.939 | 0.164 |
|  |  | kazal type 1 | 7.587 | <0.05 |
|  | Hemocytes | defensin | 0.357 | 0.707 |
|  |  | Raf | 1.991 | 0.179 |
|  |  | phenoloxidase | 0.738 | 0.499 |
|  |  | kazal type 1 | 5.357 | <0.05 |
| EFDi | Whole insect bodies | defensin | 0.117 | 0.949 |
|  |  | Raf | 2.272 | 0.119 |
|  |  | phenoloxidase | 7.248 | <0.05 |
|  |  | kazal type 1 | 3.204 | 0.052 |
|  | Midguts | defensin | 0.894 | 0.466 |
|  |  | Raf | 5.249 | <0.05 |
|  |  | phenoloxidase | 3.210 | 0.051 |
|  |  | kazal type 1 | 3.434 | <0.05 |
|  | Hemocytes | defensin | 4.511 | <0.05 |
|  |  | Raf | 0.432 | 0.733 |
|  |  | phenoloxidase | 1.688 | 0.210 |
|  |  | kazal type 1 | 1.553 | 0.240 |
| LFDi | Whole insect bodies | defensin | 0.146 | 0.931 |
|  |  | Raf | 2.041 | 0.149 |
|  |  | phenoloxidase | 0.402 | 0.754 |
|  |  | kazal type 1 | 1.600 | 0.229 |
|  | Midguts | defensin | 1.413 | 0.276 |
|  |  | Raf | 3.279 | <0.05 |
|  |  | phenoloxidase | 1.690 | 0.209 |
|  |  | kazal type 1 | 1.001 | 0.418 |
|  | Hemocytes | defensin | 1.243 | 0.327 |
|  |  | Raf | 4.004 | <0.05 |
|  |  | phenoloxidase | 0.629 | 0.607 |
|  |  | kazal type 1 | 18.866 | <0.05 |

**TABLE S5** Results of statistical analysis (ANOVA + Tukey test, P < 0.05) performed to evaluate gene expression trends over time, by comparing normalized relative quantities observed in healthy, EFDi and LFDi samples from the same treatment group (*Asaia* SF15.14Rif^R^, *Asaia* SF2.1Rif^R^, *E. coli* DH5α pKan(DsRed), and control). The expression of *defensin, Raf, phenoloxidase* and *kazal type 1 serine protease inhibitor* genes was analysed in *E. variegatus* whole insects, dissected midguts, and hemocytes. Degrees of Freedom: 2 (among-group), 12 (within-group).

| **Target gene Sample group** | **Sample type** | **Treatment** | **F-value** | **P-value** |
| --- | --- | --- | --- | --- |
| **Raf** | Whole insect bodies | Control | 0.719 | 0.507 |
|  |  | *Asaia* SF15.14Rif^R^ | 0.121 | 0.887 |
|  |  | *Asaia* SF2.1Rif^R^ | 4.764 | <0.05 |
|  |  | *E. coli* DH5α pKan(DsRed) | 0.043 | 0.958 |
|  | Midguts | Control | 0.403 | 0.677 |
|  |  | *Asaia* SF15.14Rif^R^ | 7.234 | <0.05 |
|  |  | *Asaia* SF2.1Rif^R^ | 1.680 | 0.227 |
|  |  | *E. coli* DH5α pKan(DsRed) | 0.738 | 0.498 |
|  | Hemocytes | Control | 2.693 | 0.108 |
|  |  | *Asaia* SF15.14Rif^R^ | 3.109 | 0.082 |
|  |  | *Asaia* SF2.1Rif^R^ | 0.470 | 0.636 |
|  |  | *E. coli* DH5α pKan(DsRed) | 0.442 | 0.665 |
| **kazal type 1** | Whole insect bodies | Control | 0.700 | 0.516 |
|  |  | *Asaia* SF15.14Rif^R^ | 0.288 | 0.755 |
|  |  | *Asaia* SF2.1Rif^R^ | 2.839 | 0.098 |
|  |  | *E. coli* DH5α pKan(DsRed) | 2.455 | 0.128 |
|  | Midguts | Control | 0.446 | 0.650 |
|  |  | *Asaia* SF15.14Rif^R^ | 0.192 | 0.827 |
|  |  | *Asaia* SF2.1Rif^R^ | 4.187 | <0.05 |
|  |  | *E. coli* DH5α pKan(DsRed) | 4.340 | <0.05 |
|  | Hemocytes | Control | 2.273 | 0.146 |
|  |  | *Asaia* SF15.14Rif^R^ | 8.799 | <0.05 |
|  |  | *Asaia* SF2.1Rif^R^ | 1.751 | 0.215 |
|  |  | *E. coli* DH5α pKan(DsRed) | 2.789 | 0.101 |
| **defensin** | Whole insect bodies | Control | 1.478 | 0.267 |
|  |  | *Asaia* SF15.14Rif^R^ | 3.012 | 0.087 |
|  |  | *Asaia* SF2.1Rif^R^ | 0.662 | 0.534 |
|  |  | *E. coli* DH5α pKan(DsRed) | 0.498 | 0.620 |
|  | Midguts | Control | 1.538 | 0.254 |
|  |  | *Asaia* SF15.14Rif^R^ | 1.025 | 0.388 |
|  |  | *Asaia* SF2.1Rif^R^ | 1.557 | 0.251 |
|  |  | *E. coli* DH5α pKan(DsRed) | 1.822 | 0.204 |
|  | Hemocytes | Control | 2.056 | 0.171 |
|  |  | *Asaia* SF15.14Rif^R^ | 0.762 | 0.488 |
|  |  | *Asaia* SF2.1Rif^R^ | 2.894 | 0.094 |
|  |  | *E. coli* DH5α pKan(DsRed) | 0.255 | 0.779 |
| **phenoloxidase** | Whole insect bodies | Control | 0.638 | 0.545 |
|  |  | *Asaia* SF15.14Rif^R^ | 2.050 | 0.171 |
|  |  | *Asaia* SF2.1Rif^R^ | 1.652 | 0.232 |
|  |  | *E. coli* DH5α pKan(DsRed) | 1.534 | 0.255 |
|  | Midguts | Control | 2.183 | 0.155 |
|  |  | *Asaia* SF15.14Rif^R^ | 2.906 | 0.094 |
|  |  | *Asaia* SF2.1Rif^R^ | 0.026 | 0.974 |
|  |  | *E. coli* DH5α pKan(DsRed) | 0.500 | 0.618 |
|  | Hemocytes | Control | 39.918 | <0.05 |
|  |  | *Asaia* SF15.14Rif^R^ | 0.074 | 0.929 |
|  |  | *Asaia* SF2.1Rif^R^ | 2.098 | 0.165 |
|  |  | *E. coli* DH5α pKan(DsRed) | 0.269 | 0.768 |

**TABLE S6** Adhesion values measured with crystal violet assays for trains *Asaia* SF2.1Rif^R^, *Asaia* SF15.14Rif^R^, *E. coli* DH5α pKan(DsRed), and *E. coli* ATCC 25404. The OD_610 nm_ of crystal violet stained biofilm cells was determined and subtracted to OD_610 nm_ of crystal violet stained negative controls. Error propagation was calculated by adding standard deviation of stained biofilm cells to the one of stained negative controls. Experiments were performed at 24 and 30°C and incubated for 48 and 72 hours.

|  | ***Asaia* SF2.1Rif^R^** | ***Asaia* SF15.14Rif^R^** | ***E. coli* DH5α pKan(DsRed)** | ***E. coli* ATCC 25404** |
| --- | --- | --- | --- | --- |
| **24 °C - 48 h** | 0.151 ± 0.016 | 0.040 ± 0.018 | 0.149 ± 0.037 | 0.430 ± 0.030 |
| **24 °C - 72 h** | 0.142 ± 0.022 | 0.016 ± 0.006 | 0.036 ± 0.010 | 0.813 ± 0.117 |
| **30 °C - 48 h** | 0.183 ± 0.048 | 0.063 ± 0.007 | 0.200 ± 0.034 | 1.024 ± 0.103 |
| **30 °C - 72 h** | 0.289 ± 0.153 | 0.040 ± 0.039 | 0.117 ± 0.021 | 0.620 ± 0.073 |

**SUPPLEMENTARY FIGURE LEGEND**

**FIGURE S1** Assessment of total RNA quality by electrophoresis on a denaturing agarose gel. Example of a gel showing the RNA profiles of whole *E. variegatus* body samples (1-3); midgut samples (4-6) and hemocyte samples (7-9), treated with *Asaia* SF15.14Rif^R^ and belonging to the healthy group (1, 4, 7); EFDi group (2, 5, 8), and LFDi group (3, 6, 9). RNA profiles were obtained by loading 50 ng of RNA on the denaturing gel for each sample.

**FIGURE S2.** A) Adhesion capacity of *Asaia* SF2.1Rif^R^, *Asaia* SF15.14Rif^R^, *E. coli* DH5α pKan(DsRed) and *E. coli* ATCC 25404 on a microtiter plate. Experiments were run at 24 and 30°C and incubated for 48 and 72h. n.i.: an example of negative controls (uninoculated wells). B) Partially formed ALI pellicles of *Asaia* SF15.14Rif^R^ in 2 microtiter wells.
