# Supplementary figures and images for: Activation of Immune Genes in Leafhoppers by Phytoplasmas and Symbiotic Bacteria
Source: Front Physiol. 2019 Jun 21;10:795. doi: 10.3389/fphys.2019.00795 (PMC6598074; doi:10.3389/fphys.2019.00795)

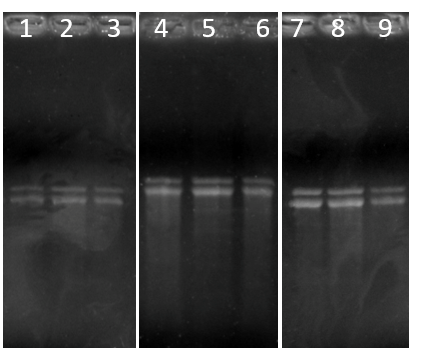

Supplement: Supplementary file 2 [file Image_1.TIF]

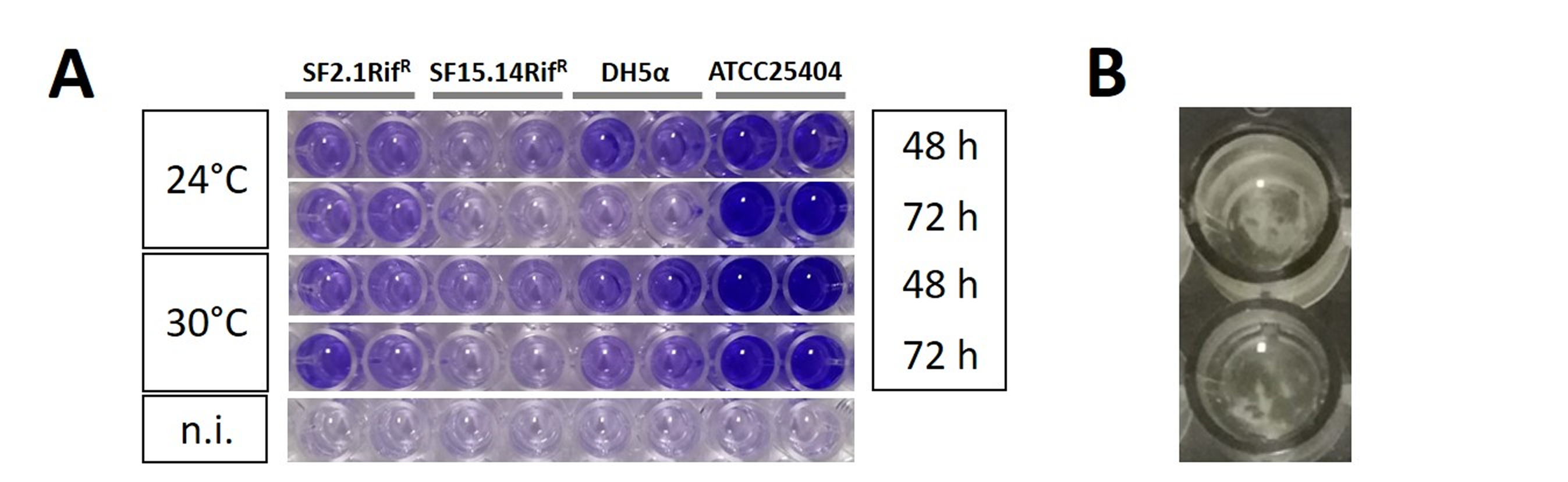

Supplement: Supplementary file 3 [file Image_2.TIF]
